# *DMD* deletions underlining mild dystrophinopathies: literature review highlights phenotype-related mutation clusters and provides insights about genetic mechanisms and prognosis

**DOI:** 10.3389/fneur.2023.1288721

**Published:** 2024-01-15

**Authors:** Fernanda Fortunato, Laura Tonelli, Marianna Farnè, Rita Selvatici, Alessandra Ferlini

**Affiliations:** Unit of Medical Genetics, Department of Medical Sciences, University of Ferrara, Ferrara, Italy

**Keywords:** dystrophinopathy, DMD, BMD, *DMD* gene, asymptomatic, hyperCKemia, mild weakness, genetic prognosis

## Abstract

*DMD* gene pathogenic variations cause a spectrum of phenotypes, ranging from severe Duchenne muscular dystrophy, the Becker milder cases, the intermediate or very mild muscle phenotypes invariably characterized by high CK, and the ultrarare fully-asymptomatic cases. Besides these phenotypes, X-linked dilated cardiomyopathy is also caused by *DMD* mutations. Males carrying *DMD* deletions with absent or very mild phenotypes have been sparsely described. We performed a horizon scan on public datasets to enroll males with the above phenotypes and carrying *DMD* deletions to delineate myopathic genotype-phenotype relationships. We inventoried 81 males, who were divided into the following clinical categorization: fully-asymptomatic males aged >43 years (A, *N* = 22); isolated hyperCKemia (CK, *N* = 35); and mild weakness (any age) with or without high CK (WCK, *N* = 24). In all cases, deleted intervals were exons 2 to 55, and no downstream exons were ever involved, apart from an exon 78 deletion in a WCK patient. All deletions were in-frame apart from the known exception to the rule of exon 2 and exon 78. We correlated the mild phenotypes (A and CK) to deleted exons, intronic breakpoints, exon-exon junctions, 3′ isoforms rule, and protein epitopes, and we found that some genetic profiles are exclusively/mainly occurring in A/CK phenotypes, suggesting they are compatible with a *quasi*-normal muscular performance. We discussed diverse pathogenic mechanisms that may contribute to mild dystrophinopathic phenotypes, and we tried to address some “critical” genetic configurations or exon content needed to preserve a semi-functional *DMD* gene.

## Introduction

Duchenne Muscular Dystrophy (DMD) is a severe X-linked recessive disorder, affecting 1 out of 5,000 males born worldwide, caused by mutations in the *DMD* gene. Located on the short arm of the X chromosome (cytogenetic location: Xp21.2-p21.1), the *DMD* gene is one of the largest known genes in the human genome, being 2.220.166 bp long, and is composed of 79 exons encoding the 427 kDa giant dystrophin protein (1).

Its protein product, the cytoskeleton protein dystrophin, is a component of the dystrophin-glycoprotein complex (DGC), a large multi-component complex with an essential role in the maintenance of the sarcolemma and mediating interactions between the cytoskeleton, membrane, and extracellular matrix (2). Loss of the DGC, caused by the absence of dystrophin, provokes a cascade of cell dysfunctions resulting in loss of physical integrity of muscle cells and contraction-induced muscle degeneration (3).

Due to the enormous size of the *DMD* gene, the mutation rate is relatively high, with approximately 1/3 of mutations occurring *de novo* and the remaining 2/3 of mutations being inherited from carrier mothers or arising from germline mosaicism (4). Mutations can be of large intragenic deletions (~65%), duplications (~10%), small mutations (~25%), and rare deep intronic mutations and complex rearrangements (< ~1%) (5).

Clinically, DMD patients manifest a progressive disease characterized by muscle mass wasting and severe weakness that starts in early childhood. Loss of independent ambulation (LoA) generally occurs around 12 years old, and cardiorespiratory failure is the main cause of death (6). In addition to progressive muscular degeneration, a DMD clinical phenotype is more often characterized by cognitive dysfunction, neuropsychological problems (anxiety, depression, and emotional disturbance), and neurobehavioral abnormalities (autism spectrum, attention-deficit hyperactivity disorder, and obsessive-compulsive disorder) (7).

The milder, allelic form of the condition, Becker muscular dystrophy (BMD), presents, like DMD, with a predominant proximal distribution of muscle weakness and wasting; however, the course is more benign and heterogeneous with a wide spectrum of clinical presentations that range from delayed loss of independent ambulation to almost asymptomatic cases with only elevated activity of creatine kinase (CK).

Serum CK is commonly used as a screening biomarker to detect patients with suspected dystrophinopathies early. However, it may not be optimal in monitoring disease progression and response to therapy; indeed, its serum levels greatly vary depending on sarcolemma damage, remaining muscle mass, environmental factors (such as metabolic changes, muscle trauma, and exercise), and aging. Besides CK, muscle-injury proteins specifically expressed in other tissues (e.g., the heart) could be useful in providing information about cardiac involvement. Particularly among these, cardiac troponin I (TNNI3) and Interleukin 1 Receptor-Like 1 Protein (ST2), being associated with cardiac degeneration, are potential biomarkers for cardiac injury (8).

Indeed, a common complication of both DMD and BMD is dilated cardiomyopathy (DCM), the severity of which may also depend on mutation type and location (9). Very mild or even asymptomatic individuals carrying *DMD* mutations are singularly reported in the literature and only a few papers have attempted a more systematic case review.

Waldrop et al. conducted a review of patients with dystrophinopathy in published literature and unpublished databases to define phenotypic features of patients with exon 51 “skip-equivalent” deletions, identifying a wide phenotypic variability which included asymptomatic patients, isolated hyperCKemia, and isolated dilated cardiomyopathy (10).

An integrated diagnostic workflow in an Italian multicenter study evaluating patients with asymptomatic or minimally symptomatic hyperCKemia has led to the identification of three male patients carrying pathogenic variants in the *DMD* gene (11).

Tuffery-Giraud et al. described data from the French UMD–DMD database including *DMD* molecular defects and clinical characteristics of 2405 patients with dystrophinopathy at a nationwide level: only one asymptomatic male patient was identified (12).

Recently, an extensive systematic review of genotype-phenotype correlations in patients with dystrophinopathy was performed in order to identify phenotypic severity predictors to provide clinicians with information about disease progression (13).

To review these atypical cases, we performed a horizon scan on public datasets, including PubMed and LOVD, and also added our internal patient series of 1,200 males carrying a *DMD* mutation to delineate genotype-phenotype relationships. We inventoried 81 males, and in order to define genotype-phenotype correlation, we proposed the following clinical categorization: (A) = fully-asymptomatic males aged >43 years; (CK) = isolated hyperCKemia; and (WCK) = mild weakness (any age) with or without high CK. The results are shown in this review with consideration to dystrophinopathy pathogenesis and roles in the phenotype spectrum some critical exons may play.

## Literature search and clinical categorization

Based on a literature horizon scan, we inventoried all male cases reported to be carrying a *DMD* deletion and with a non-DMD/BMD phenotype. A literature search in PubMed (14) was conducted using the following queries: “*DMD*” AND “Asymptomatic” OR “HyperCKemia” OR “Mild phenotype”. Another search was performed in the LOVD *DMD* database (15) by selecting patients from the “hCK” category.

This literature search led to the identification of patients by published papers and reports; the internal database including 1,200 DMD diagnoses was also consulted.

The identified patients were classified into the following subgroups: fully-asymptomatic patients >43 years old (A); asymptomatic patients with hyperCKemia (CK); and patients with mild muscle phenotype (weakness, calf hypertrophy, or muscle cramps) with or without hyperCKemia (WCK).

To enroll asymptomatic adults, we decided to adopt an age cut-off of >43 years which is based on literature data since all asymptomatic individuals reported in the landscaped articles were aged 43 or more.

### Patients' cohort

Based on our literature horizon scan, we were able to identify 81 patients divided into the following clinical categories: 22 A, 35 CK, and 24 WCK. Clinical phenotypes and genetic findings of the identified patients were derived from 11 papers and 16 reports: in detail, 49 patients were described in original research and 29 patients in reports. Three patients' data were also collected from the internal database of our Medical Genetics Unit.

Patients' clinical phenotypes, genetic findings, and literature references are reported in detail in Supplementary Table 1.

### A, CK, and WCK patients: protein topography of *DMD* mutations

Full-length dystrophin is composed of four major domains, including the N-terminal F-actin-binding domain (ABD; encoded by exons 1–8), rod (R; encoded by exons 8–64), cysteine-rich (CR; encoded by exons 64–70), and C-terminal (CT; encoded by exons 71–79) domains.

The rod domain can be further divided into 24 spectrin-like repeats (R1-R24) and four interspersed hinges (H1-H4) (16). These hinge regions are found at positions within the protein encoded by exon 9 and part of exon 8 (hinge 1), exon 17 (hinge 2), and exon 50 to 51 (hinge 3); the hinge 4 sequence has not been definitively identified (17).

The cysteine-rich domain consists of subdomains WW (tryptophan-rich domain), EFH1, EFH2 (EF hand domains 1 and 2), and ZZ (zinc finger domain) (18).

In asymptomatic males of our cohort, deleted exons involved R17-22 repeats (encoded by exons 45–55) and the hinge 3 domain only; hinge 1 and hinge 2 were never involved in this group of patients.

While hinge 1 involvement was only found to be associated with the CK phenotype, deletions clustering in the hinge 2 region were identified in both CK and WCK patients.

As in asymptomatic patients, deleted exons in CK and WCK phenotypes mainly involved R17-R22 repeats.

Within the deletions identified in all considered phenotypes (A, CK, and WCK), none involved hinge 4; similarly, EFH1, EFH2, WW, ZZ domains, and the C-terminus translation were preserved in all identified patients since all deleted intervals clustered between exons 2 to 55 (apart from an exon 78 deletion in a WCK patient).

### A, CK, and WCK patients: distribution of *DMD* mutations

In the *DMD* gene, mutations tend to cluster within two major hot-spot regions with small differences within the different patients' populations: the region of exons 2–20, where mutations remove some or all of the actin-binding sites together with a part of the rod domain (19), and the region of exons 44–55, where mutations remove part of the rod domain which is essential for the correct localization of nNOS at the sarcolemma (20).

Among patients identified with A, CK, and WCK phenotypes, deletions were heterogeneous and substantially overlapped those already published in other patients' cohorts with some peculiarities. Indeed, in all cases of our 81 patients' cohort, deleted intervals clustered between exons 2 and 55, and downstream exons were never involved apart from one patient carrying an exon 78 deletion (exception-to-the-rule).

Both single and multiple exon deletions were observed in our cohort of patients. Single exon deletions were found in 13 patients and reported in exon 2 (*N* = 1), 16 (*N* = 1), 24 (*N* = 1), 26 (*N* = 2), 48 (*N* = 7), and 78 (*N* = 1); the most common single exon deletion occurred in exon 48, having been identified in seven patients.

Multiple exon deletions were reported in 68 patients and varied greatly. Among them, deletion 45–55 was the most frequent type, occurring in 17/68 patients.

Distributions of deletions in our cohort thus confirm the well-known distal hot spot located in the broader region at the 3′ end of the *DMD* gene, involving exons 45 to 55. All deletions identified in our cohort were in-frame apart from the known exception to the rule of exon 2 and exon 78, which are out-of-frame, though causing mild phenotypes (21, 22).

The distribution of *DMD* deletions in patients with A, CK, and WCK phenotypes is represented in Supplementary Table 2.

### A, CK, and WCK patients: genotype-phenotype correlation

Patients in category A showed deletions involving exon 2 (*N* = 1), 2–7 (*N* = 1), 16 (*N* = 1), 38–44 (*N* = 2), 45–51 (*N* = 1), 45–55 (*N* = 6), 48 (*N* = 1), 48–51 (*N* = 3), 48–53(*N* = 1), 49–51 (*N* = 1), 50–51 (*N* = 2), and 51–52 (*N* = 2).

In categories CK and WCK, deletions were quite heterogeneous although deletions 45–51 (20.3%) and 45–55 (18.6%) were the most frequent.

While deletions of exons 50–51 and 51–52 were identified only in asymptomatic individuals, deletions of exons 45–55 occurred in all phenotypes (A, CK, and WCK).

This in-frame deletion in the dystrophin central domain has been described in the literature in asymptomatic subjects and patients with more severe muscular involvement or presenting with significant cardiomyopathy (23–26).

Patients of our cohort substantially overlap this clinical heterogeneity, being deletions of exon 45–55 reported in all considered phenotypes (A, CK, and WCK).

**Figure 1:**
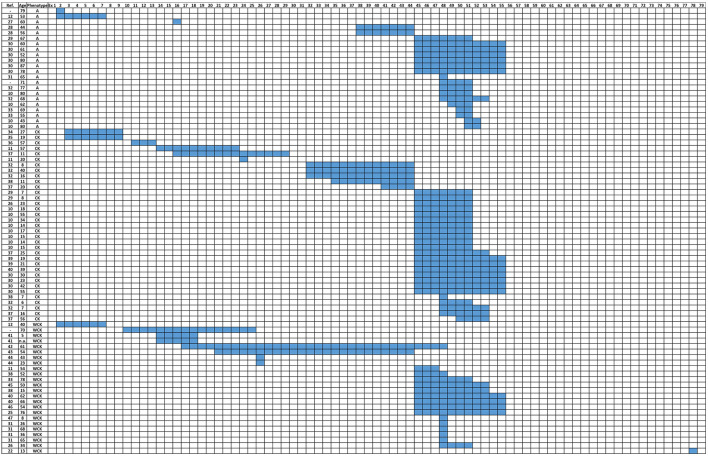
Distribution of DMD deletions according to patients' phenotypes (A, CK, and WCK). A, Asymptomatic; CK, Isolated HyperCKemia; WCK, mild weakness with or without high CK, (-): individuals/patients from the internal database of the UNIFE Medical Genetics Unit.

The distribution of *DMD* deletions according to A, CK, and WCK phenotypes is reported in Figure 1 (27–47).

## Discussion

Although the frame rule (and its exceptions) explains the genotype-phenotype relationship in the majority of *DMD* gene pathogenic variations (mutations) causing Duchenne and Becker muscular dystrophies, intermediate phenotypes, and isolated cardiomyopathy (XLDC), the etiopathogenesis of sparse and rare asymptomatic or non-classic dystrophic cases remains largely unexplained. Understanding the mechanisms underlining these rare dystrophinopathies is challenging since it would impact many medical aspects. Therefore, we have reviewed all known published/public cases carrying *DMD* deletions and have tried to identify common genetic configurations, relationships between deletions and loss/maintaining of protein domains, the topography of intronic breakpoint deletions, and novel exon junctions of the resulting transcripts. It undoubtedly would have been useful to consider all mutation types; however, a systematic, *in silico* analysis of duplications or small variations, whose impact on phenotype is not unequivocal and could be influenced by other factors (5), might be difficult and likely not produce consistent relationships with the phenotype.

### Genetic prognosis and clinical validity

Predicting the pathogenic meaning of incidental findings in the *DMD* gene often coming from prenatal comparative genomic hybridization (CGH) testing is important. CGH array has become a routine exam during pregnancy, both as a screening procedure and in cases where fetal echography anomalies may occur (4). Therefore, *DMD* deletions can be identified in prenatal testing in couples with no DMD recurrence.

It is to be noted that, in our revised cohort, deletions of exons 50–51 and 51–52 occur in category A only. Deletions of exon 50–51 are described in BMD although the clinical phenotype generally ranges from very mild to asymptomatic (10, 33, 48). This deletion belongs to the so-called “skip-51 equivalent” class, which has been clinically investigated to predict the phenotype resulting from the exon 51 (eteplirsen drug) skipping. CGH outputs such as exon 50–51 deletions should be considered very mild phenotypes, as already noted (10). The deletion of exon 51–52 occurs only in category A as well, but it is a rare deletion found in Caucasian populations and described in only one BMD patient in China (5, 49). Our analysis strongly supports that the 51–52 deletion may be “purely asymptomatic and non-dystrophic”, thus orienting genetic counseling in case of prenatal incidental findings toward a benign phenotype. The very rare, isolated exon 2 deletion was extensively studied as a non-dystrophic mutation since it associates with the activation of an alternative ATG in exon 6 (21). An exon 16 deletion is also rare and invariably associated with an asymptomatic phenotype (27); therefore, it is also to be considered a “benign” non-dystrophic variant.

### Genotype data and clinical implications in DMD

In all of our cases, we observed that deleted intervals were exons 2 to 55, and no downstream exons were ever involved apart from an exon 78 deletion (exception-to-the-rule) in a WCK patient. Obviously, domains downstream exon 55 are intact in the A, CK, and WCK categories and preserve H4, EFH1, EFH2, WW, and ZZ domains and the C-terminus translation. These data also imply that the *DMD* 3′ Dp71 isoform is never involved and is always preserved in these phenotypes. This reinforces their importance in the correct development of muscle function, as described (50). It is to be noted that the 56–79 region corresponds to the ancient sea urchin *DMD* gene, whose function is purely annelid-striated muscle-related, supporting our observation and consequent hypothesis that this region is very relevant for muscle function. Deletions in category A (*N* = 22) were exon 2 (*N* = 1), 2–7 (*N* = 1), 16 (*N* = 1), 38–44 (*N* = 2), 45–51 (*N* = 1), 45–55 (*N* = 6), 48 (*N* = 1), 48–51 (*N* = 3), 48–53 (*N* = 1), 49–51 (*N* = 1), 50–51 (*N* = 2), and 51–52 (*N* = 2). Deletions in the CK and WCK categories vary, where deletion 45–51 represents 20.3% (12/59) of cases and deletion 45–55 accounts for 18.6% (11/59). Single exon deletions were exon 2, 16, 24, 26, 48, and 78 in A, CK, and WCK patients. All deletions were in-frame apart from the known exception of exon 2 and exon 78. The isolated exon 48 deletion is intriguing since it occurs in two mild cases (A and CK) as well as in five WCK individuals (Supplementary Table 2). Exon 48 deletion was studied in many papers and was reported as a “cardiac deletion” (51). This deletion is predicted to create a new hinge domain adjacent to the natural hinge 3, which may heavily impact the protein structure. Indeed, its occurrence in all mild phenotypes, including some young patients (category WCK), makes its interpretation difficult. This deletion deserves to be studied using genome sequencing techniques (see below) to define the intronic breakpoints and/or eventually concurring complex rearrangements which may modulate its effect.

**Figure 2:**
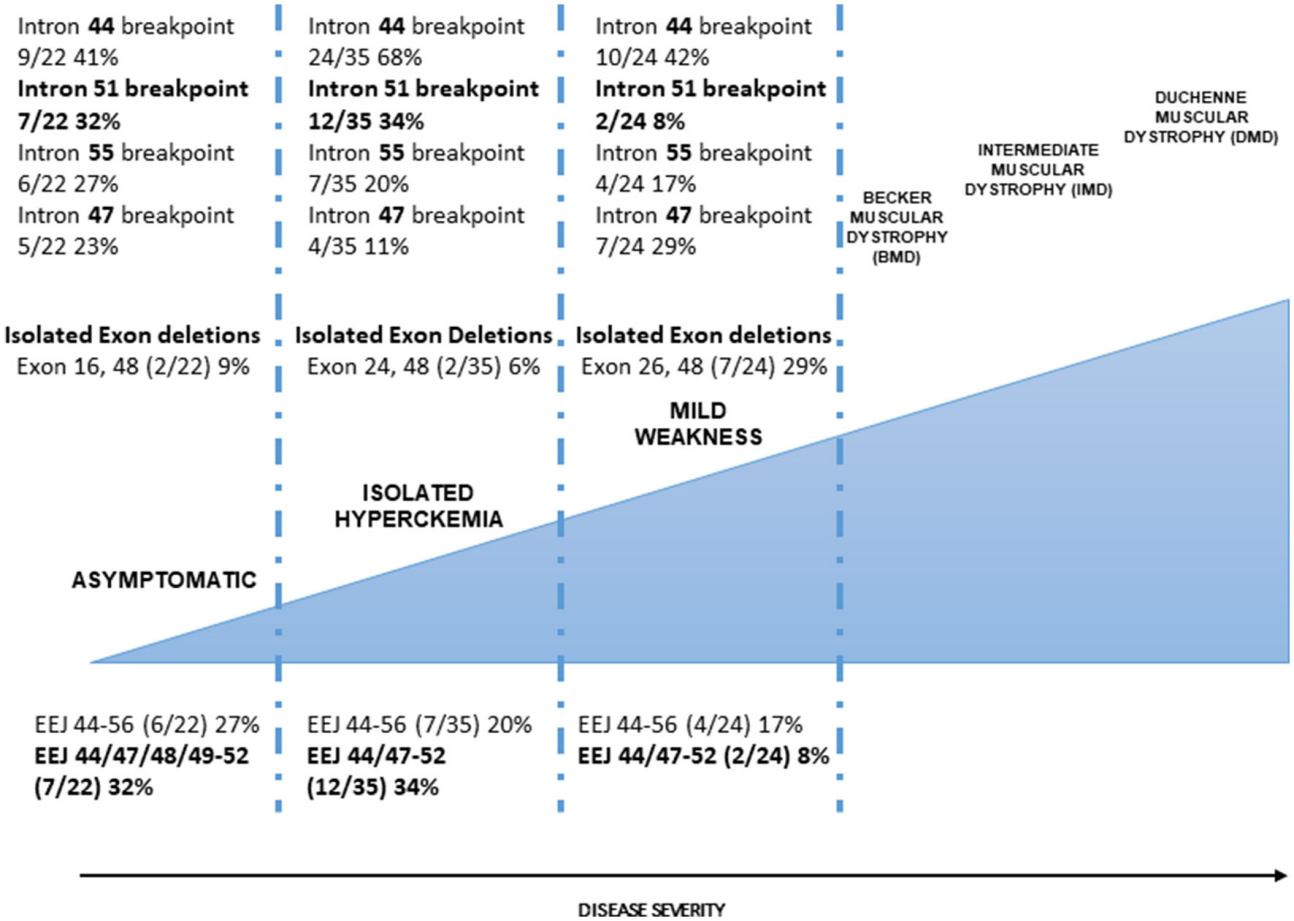
Dystrophinopathies clinical severity and correlation with the genetic profile. The correlation between intronic breakpoints. Introns 44 (major break site in the DMD 3′ mutation hot spot), 47, and 55 breakpoints occur with similar frequency in the three categories (A, CK, and WCK) although some differences can be seen but with no significant differences; intron 51 breakpoint occurs with high frequency in A and CK categories. This difference is statistically significant if categories A and CK are merged compared to category WCK. The occurrence of isolated exon deletions. In-frame isolated exon deletions are rare in milder (A and CK) categories, being about 10% of all deletions; WCK single-exon deletions (26 and 48) represent 29% of all deletion events, suggesting that single-exon deletions, despite causing a minimal loss of protein domain, are not associated with a milder phenotype. The correlation between the exon-exon junctions. Exon-exon junctions (EEJs) are important for the correct splicing process of the DMD transcript. In our cohorts, the 44–56 EEJ is common to all categories although occurring in different frequencies with a gradient associated with the disease severity (more frequent in A phenotype). EEJ 44/47–52 is mainly present in the A and CK phenotypes. Interestingly, this EEJ invariably implies the presence of the 3′ deletion breakpoint in intron 51 which determines the loss of exon 51.

We attempted a correlation between introns, where at least one breakpoint occurs, and the phenotype (Figure 2). Intron 44, as expected, is the most frequent breakpoint site in all of the categories (A: 41%, CK: 68%, and WCK: 42%). Similarly, occurring breakpoints are in introns 55 and 47, but not in intron 51 where the major breakpoints occur in 32% of category A, 34% of category CK, and only 8% in category WCK. Obviously, these differences may reflect the deletion intervals in categories A and CK, where we have already noted that deletions with a major breakpoint in intron 51 (the so-called 51 skip equivalent) are much more frequent than in other cases. Intron 51, again, would deserve to be fully characterized in these patients since it possibly provides regulatory motifs that impact dystrophin protein synthesis efficiency (see also below).

Many deletions overlap among A, CK, and WCK categories, especially those occurring at the exon intervals 45–51, and it is not possible to identify other phenotype-specific deletion profiles.

### Protein epitopes' impact and dystrophin muscular function

In asymptomatic males, deleted exons involved R17-22 repeats and hinge 3 and 4 domains only; other repeats, hinge 1 and hinge 2, were never involved. The dispensability of spectrin repeats is well-known in DMD since it is frequently missed in the BMD phenotype. The four dystrophin hinge domains influence muscle maturation and maintenance (52). Hinges 2 and 4 are particularly important for the sarcolemma complex since they contain a polyproline site and a WW motif, respectively, and are required for binding to beta-dystroglycan. Our review suggests that keeping hinge 1 and 2 domains might be sufficient to preserve intact muscle functions (see below for the impact on gene therapy).

### Impact on protein synthesis efficiency

Different dystrophin synthesis efficiency depending on different *DMD* deletions has already been observed (26). In this study, 17 BMD patients carrying in-frame deletions were correlated to the protein levels. All patients showed 44% of control dystrophin levels; however, BMD patients with deletions up to exon 51 showed significantly higher dystrophin levels. This supports our observation and hypothesis that exon 51 “skip-equivalent” deletions (45–51 or 50–51) and deletion 51–52 can truly allow the production of more dystrophin protein. The reasons for that are fully unknown although the finding of the high frequency of an intron 51 breakpoint in these individuals is intriguing. As already proposed, these deletions should be studied at the genomic level, and breakpoints should be defined in order to unravel possible regulatory mechanisms.

### Impact of therapy design

As we have suggested above, preservation of hinge 1 and 2 domains might be sufficient to keep a “quasi-normal” muscle function. *DMD* gene therapy is already in clinical trials or provisionally approved by the FDA (53). The three mini-dystrophin constructs used for gene therapy are different in terms of exon content. Interestingly, the Sarepta (Genethon) construct includes both H1 and H2 (and H4), while other constructs do not (54). The invariable conservation of H1 and H2 in all asymptomatic individuals of our cohort may suggest that these domains are crucial for correct muscle function via the dystrophin interactions at the sarcolemma.

Constructs designed for gene therapies also raise the issue of “critical exons”. The concept of critical exons might be related to protein domains (as for hinge domains) or to specific protein regions that are dispensable (spectrin-like repeats 17–22) or not (COOH Terminus). Another view for looking at critical exons is to consider exon-exon junctions. RNA exon-exon junctions (EEJs) are formed via the exon junction complex (EJC) which recognizes the appropriate EEJ via the messenger ribonucleoprotein (mRNP) component in order to facilitate correct splicing. Correct EEJs assembled in EJCs accompany mRNA during its export from the nucleus into the cytoplasm since they communicate information about the splicing process and the position of introns. In addition, an EJC's core component and its associated proteins regulate different steps of gene expression, including translation efficiency and nonsense-mediated mRNA decay (NMD) (55, 56).

To provide a relevant link to the sarcolemma and muscle cells, the EJC is involved in the cardiac myocyte stress response (57). Unnatural DMD exon-exon junctions created by deletion events may impact all of the abovementioned functions and may account for the different clinical effects we observe in patients possibly related to correct and efficient splicing or protein synthesis rate. Looking at our cohort, we can also see that, exactly overlapping the breakpoint data, EEJ 44/47–52 frequency is very different in categories A and CK (18 and 34%, respectively) compared to category WCK (8%) (Figure 2). These EEJs reflect the exon 51 deletion and the intron 51 breakpoint, so the data are obviously confirmatory; nevertheless, they again underline the need to get insights into intron 51, its unnatural EEJs, and its sequence.

### Reflections and future research avenues

As an outcome of this work, we may propose some “statements” to address possible criticisms and decisions during the diagnostic practice (Table 1). Indeed, our knowledge about why some *DMD* deletions cause no or mild muscle phenotypes is still poor. Therefore, we have drafted two main “recommendations” which we believe to be important in view of having DMD patients in trials or under therapy using RNA molecules or transgenes.

**Table 1:** Relevant reflections as take-home messages following reviewing asymptomatic or mild DMD cases.

**Statement**	**Rationale**
The DMD 3′ region (exon 56–79) is important for the muscle function	Invariably preserved in A and CK phenotypes
Hinges 1 and 2 are important for preserving the muscle function	Invariably preserved in A phenotype
Deletions of exons 2 and 16 are “non dystrophic” mutations	Invariably associated to asymptomatic cases
Deletion of exon 51–52 are causing extremely mild cases	Invariably associated to extremely mild/asymptomatic cases
Isolated exon 48 has an unpredictable phenotype	Identified in only one A and one CK individual, but frequent (5/24) in patients with WCK
Isolated, in-frame, exon deletions do not predict a mild or asymptomatic phenotype	Isolated exon deletions occur in 7% of A/CK cases and 29% of WCK cases

1) Intronic breakpoints should be finely characterized. Whole genome sequencing (WGS) is now a standard procedure included in routine diagnostics, and its cost is greatly decreased (58). It would not be too expensive or time-consuming to include WGS analysis in cases where the genotype-phenotype is not coherent with the current knowledge or frame rule or in cases of patients enrolled in clinical trials. DMD knowledge would greatly benefit this approach, certainly providing numerous amounts of data concerning patients' true genotypes and likely allowing the identification of novel, non-coding, regulatory motifs impacting clinical features.

2) 3′ isoform preservation/loss should be studied. Identical to the previous recommendation, 3′ isoform profiling should be performed again in some patients with a lack of genotype-phenotype correlation as well as in boys enrolled in trials. *DMD* 3′ isoforms, especially Dp71 (as we discussed above) can play a role in dystrophin production, such as quantity and quality, and in developmental perspective.

Thus, WGS analysis coupled with non-invasive *DMD* isoforms' transcription studies (59) is fully feasible and not too expensive, especially if applied to smaller sub-cohorts of patients.

## Conclusions

We hope the readers have found this mini-review interesting and useful. The take-home message is that asymptomatic phenotypes may provide clues in understanding DMD etiopathogenesis, in better defining the complex dystrophin protein transcription, translation, and regulatory paths, and in possibly refining therapy design. We have highlighted that some deletions should be carefully considered when identified as incidental findings and genetic counseling must be always offered to help the interpretation of these rare DMD genotypes.

## Author contributions

FF: Data curation, Formal analysis, Investigation, Methodology, Supervision, Validation, Writing—original draft. LT: Data curation, Formal analysis, Investigation, Methodology, Writing—original draft. MF: Formal analysis, Investigation, Methodology, Resources. RS: Data curation, Investigation, Supervision, Validation. AF: Conceptualization, Formal analysis, Funding acquisition, Resources, Supervision, Validation, Visualization, Writing—original draft, Writing—review & editing.
