# Supplementary material for: DMD deletions underlining mild dystrophinopathies: literature review highlights phenotype-related mutation clusters and provides insights about genetic mechanisms and prognosis
Source: Front Neurol. 2024 Jan 15;14:1288721. doi: 10.3389/fneur.2023.1288721 (PMC10823016; doi:10.3389/fneur.2023.1288721)
Supplement: Supplementary file 1 [file Table_1.docx]

**Supplementary Table 1**. Genetic and clinical details of A, CK and WCK patients. A = Asymptomatic, CK = Isolated HyperCKemia, WCK = mild weakness with or without high CK, n.a = not available, in the last right column we reported the exon which skipping is induced using the corresponding antisense oligonucleotide, thus generating the “skip equivalent transcript” following exon skipping therapy approach. We considered only published antisense oligonucleotide compounds in clinical trials only.

| **Citation Literature**  **full reference** | **Patient age at genetic diagnosis** | **Phenotype**  **Category** | **CK value (U/l)** | **DMD deletion** | **Muscle condition information** | **Cardiac condition information** | **Exon to be skipped to get a “skip equivalent”** |
| --- | --- | --- | --- | --- | --- | --- | --- |
| **INTERNAL DATASET** | 79 | A | n.a. | Ex 2 | Asymptomatic | n.a. | n.a. |
| **INTERNAL DATASET** | 70 | WCK | n.a. | Ex 10-25 | Mild weakness | n.a. | n.a. |
| **INTERNAL DATASET** | 71 | A | n.a | Ex 48-51 | Asymptomatic | n.a. | 51 |
| Anthony K et al. Dystrophin quantiﬁcation and clinical correlations in Becker muscular dystrophy: implications for clinical trials. Brain (2011) 134: 3547-59  **PAPER** | 23 | CK | High CPK | Ex 45-51 | Asymptomatic | Negative | 45/51 |
|  | 34 | WCK | High CPK | Ex 48-51 | Cramps | Negative | 51 |
| Beggs AH et al.  Exploring the molecular basis for variability among patients with Becker muscular dystrophy: dystrophin gene and protein studies. Am J Hum Genet (1991) 49:54–67  **PAPER** | 11 | CK | High CPK | Ex 35-44 | Asymptomatic | n.a. | 44 |
|  | 52 | WCK | n.a. | Ex 45-48 | Weakness | n.a. | 45 |
|  | 15 | WCK | n.a. | Ex 45-53 | Calf hypertrophy | n.a. | 45/53 |
|  | 7 | CK | High CPK | Ex 48 | Asymptomatic | n.a. | n.a. |
| Bosone I et al.  Late onset and very mild course of Xp21 Becker type muscular dystrophy. Clin Neuropathol (2001) 20:196–9  **REPORT** | 50 | WCK | 200 | Ex 45-53 | Calf hypertrophy | Negative | 45/53 |
| Collins AL et al.  An inherited dystrophin deletion without muscle weakness. J Med Genet. (1994) 31:505  **REPORT** | 5 | WCK | 7854 | Ex 14-18 | Occasional muscle cramps | n.a. | n.a. |
|  | n.a. (maternal grandfather) | WCK | 520 | Ex 14-18 | Occasional myalgia | n.a. | n.a. |
| Comi GP et al.  Clinical variability in Becker muscular dystrophy. Genetic, biochemical and immunohistochemical correlates. Brain (1994) 117:1–14  **PAPER** | 11 | CK | High CPK | Ex 16-29 | Asymptomatic | n.a. | n.a |
|  | 20 | CK | High CPK | Ex 41-44 | Asymptomatic | n.a. | 44 |
|  | 25 | CK | High CPK | Ex 45-53 | Asymptomatic | n.a. | 45/53 |
|  | 56 | CK | High CPK | Ex 50-53 | Asymptomatic | n.a. | 53 |
|  | 16 | CK | High CPK | Ex 48-53 | Asymptomatic | n.a. | 53 |
| Ferreiro V et al. Asymptomatic Becker muscular dystrophy in a family with a multiexon deletion. Muscle Nerve (2009) 39: 239–43  **REPORT** | 19 | CK | 849 | Ex 45-55 | Asymptomatic | n.a. | 45 |
|  | 21 | CK | 978 | Ex 45-55 | Asymptomatic | n.a. | 45 |
| Gemelli C et al.  An integrated approach to the evaluation of patients with asymptomatic or minimally symptomatic hyperCKemia. Muscle Nerve (2022) 65:96-104  **PAPER** | 57 | CK | High CPK | Ex 14-23 | Asymptomatic | n.a. | n.a. |
|  | 20 | CK | High CPK | Ex 24 | Asymptomatic | Negative | n.a. |
|  | 54 | WCK | n.a. | Ex 45-47 | Weakness | Negative | 45 |
| Helderman-van den Enden AT et al. Becker muscular dystrophy patients with deletions around exon 51; a promising outlook for exon skipping therapy in Duchenne patients.Neuromuscular Disorders 20 (2010) 251–4  **REPORT** | 78 | WCK | n.a. | Ex 45-51 | Cramps | Negative | 45/51 |
|  | 69 | A | n.a. | Ex 50-51 | Asymptomatic | Negative | 51 |
|  | 55 | A | n.a. | Ex 50-51 | Asymptomatic | n.a. | 51 |
| Melis M A et al.  Elevation of serum creatine kinase as the only manifestation of an intragenic deletion of the dystrophin gene in three unrelated families.[Eur J Paediatr Neurol.](https://www.ncbi.nlm.nih.gov/pubmed/10726828)(1998) 2:255-61  **REPORT** | 8 | CK | 5382 | Ex 32-44 | Asymptomatic | n.a. | 44 |
|  | 40 | CK | 290 | Ex 32-44 | Asymptomatic | n.a. | 44 |
|  | 16 | CK | 314 | Ex 32-44 | Asymptomatic | n.a. | 44 |
|  | 6 | CK | 2900 | Ex 48-51 | Asymptomatic | n.a. | 51 |
|  | 77 | A | 130 | Ex 48-51 | Asymptomatic | n.a. | 51 |
|  | 7 | CK | 1200 | Ex 48-53 | Asymptomatic | n.a. | 53 |
|  | 68 | A | n.a. | Ex 48-53 | Asymptomatic | n.a. | 53 |
| Morrone A et al. Asymptomatic dystrophinopathy. Am J Med Genet (1997) 69:261–7  **REPORT** | 8 | WCK | 2835 | Ex 48 | Calf hypertrophy | Negative | n.a. |
| Nakamura A et al. Deletion of exons 3-9 encompassing a mutational hot spot in the DMD gene presents an asymptomatic phenotype, indicating a target region for multiexon skipping therapy. J Hum Genet (2016) 61:663–7  **REPORT** | 27 | CK | 217-1474 | Ex 3-9 | Asymptomatic | Negative | n.a. |
| Palmucci L et al.  Unusual expression and very mild course of Xp21 muscular dystrophy (Becker type) in a 60‐year‐old man with 26 percent deletion of the dystrophin gene.  Neurology (1994) 44 (3 Part 1):541-3  **REPORT** | 54 | WCK | 938 | Ex 21-44 | Mild proximal weakness | Negative | 44 |
| Saengpattrachai M et al. Grandpa and I have dystrophinopathy?:  Approach to asymptomatic  hyperCKemia. Pediatr Neurol (2006) 35:145-9  **REPORT** | 8 | CK | 2950 | Ex 45-51 | Asymptomatic | Negative | 45/51 |
|  | 7 | CK | 896 | Ex 45-51 | Asymptomatic | Negative | 45/51 |
|  | 67 | A | 63 | Ex 45-51 | Asymptomatic | Negative | 45/51 |
| Taglia A et al.  Clinical features of patients with dystrophinopathy sharing the 45-55 exon deletion of DMD gene.Acta Myologica (2015) 34: 9-13  **PAPER** | 39 | CK | High CPK | Ex 45-55 | Asymptomatic | Negative | 45 |
|  | 62 | WCK | High CPK | Ex 45-55 | Calf hypertrophy | Negative | 45 |
|  | 66 | WCK | High CPK | Ex 45-55 | Calf hypertrophy | Negative | 45 |
| Toksoy G et al.  Mutation spectrum of 260 dystrophinopathy patients from Turkey and important highlights for genetic counseling.Neuromuscular Disorders 29 (2019) 601-13  **PAPER** | 19 | CK | 721 | Ex 3-9 | Asymptomatic | Negative | n.a. |
| Tselikas L et al.  Late onset Becker muscular dystrophy. A case report and literature review.  Rev Med Interne. (2011) 32:181-6  **REPORT** | 54 | WCK | 3196 | Ex 45-55 | Calf hypertrophy | Negative | 45 |
| Vandenhende MA et al. Dilated cardiomyopathy and lipid-lowering drug muscle toxicity revealing late-onset Becker’s disease. Rev Med Interne (2005) 26:977–9  **REPORT** | 57 | CK | 350 | Ex 11-13 | Asymptomatic | n.a. | n.a. |
| Zimowski JG et al.  A rare subclinical or mild type of Becker muscular dystrophy caused by a single exon 48 deletion of the dystrophin gene. J Appl Genet (2017) 58(3):343-7  **PAPER** | 65 | A | n.a. | Ex 48 | Asymptomatic | n.a. | n.a. |
|  | 36 | WCK | n.a. | Ex 48 | Cramps | n.a. | n.a. |
|  | 26 | WCK | High CPK | Ex 48 | Cramps and calf hypertrophy | n.a. | n.a. |
|  | 68 | WCK | High CPK | Ex 48 | Calf hypertrophy | n.a. | n.a. |
|  | 65 | WCK | n.a. | Ex 48 | Calf hypertrophy | n.a. | n.a. |
| Waldrop MA et al. Clinical Phenotypes of DMD Exon 51 Skip Equivalent Deletions: A Systematic Review. J Neuromuscul Dis. (2020) 7:217-229  **PAPER** | 80 | A | Normal | Ex 48-51 | Asymptomatic | Negative | 51 |
|  | 62 | A | Normal | Ex 49-51 | Asymptomatic | Negative | 51 |
|  | 43 | A | Normal | Ex 51-52 | Asymptomatic | Negative | 51 |
|  | 80 | A | Normal | Ex 51-52 | Asymptomatic | Negative | 51 |
|  | 34 | CK | High CPK | Ex 45-51 | Asymptomatic | Negative | 45/51 |
|  | 14 | CK | High CPK | Ex 45-51 | Asymptomatic | Negative | 45/51 |
|  | 17 | CK | High CPK | Ex 45-51 | Asymptomatic | Negative | 45/51 |
|  | 14 | CK | High CPK | Ex 45-51 | Asymptomatic | Negative | 45/51 |
|  | 15 | CK | High CPK | Ex 45-51 | Asymptomatic | Negative | 45/51 |
|  | 15 | CK | High CPK | Ex 45-51 | Asymptomatic | Negative | 45/51 |
|  | 18 | CK | High CPK | Ex 45-51 | Asymptomatic | Negative | 45/51 |
|  | 55 | CK | High CPK | Ex 45-51 | Asymptomatic | Negative | 45/51 |
| Poyatos-García J et al. Dystrophinopathy Phenotypes and Modifying Factors in DMD Exon 45-55 Deletion. Ann Neurol. (2022) 92:793-806  **PAPER** | 60 | A | Normal | Ex 45-55 | Asymptomatic | Negative | 45 |
|  | 61 | A | Normal | Ex 45-55 | Asymptomatic | Negative | 45 |
|  | 52 | A | Normal | Ex 45-55 | Asymptomatic | Negative | 45 |
|  | 80 | A | Normal | Ex 45-55 | Asymptomatic | Negative | 45 |
|  | 87 | A | Normal | Ex 45-55 | Asymptomatic | Negative | 45 |
|  | 78 | A | Normal | Ex 45-55 | Asymptomatic | Negative | 45 |
|  | 30 | CK | 780 | Ex 45-55 | Asymptomatic | Negative | 45 |
|  | 23 | CK | 600 | Ex 45-55 | Asymptomatic | Negative | 45 |
|  | 42 | CK | 7000 | Ex 45-55 | Asymptomatic | Negative | 45 |
|  | 55 | CK | 900 | Ex 45-55 | Asymptomatic | Negative | 45 |
| England SB et al.  Very mild muscular dystrophy associated with the deletion of 46% of dystrophin. Nature (1990) 343(6254):180-2  **REPORT** | 61 | WCK | n.a. | Ex 17-48 | Weakness | n.a. | n.a. |
| Tuffery-Giraud S et al. Genotype-phenotype  analysis in 2,405 patients with a dystrophinopathy using the  UMD-DMD database: A model of nationwide knowledgebase.  Human Mutation (2009) 30: 934–45  **PAPER** | 40 | WCK | n.a. | Ex 2-7 | Weakness | n.a. | n.a. |
|  | 53 | A | n.a. | Ex 2-7 | Asymptomatic | n.a. | n.a. |
| Nakamura A et al.  Follow-up of three patients with a large in-frame deletion of exons 45-55 in the Duchenne muscular dystrophy (DMD) gene. J Clin Neurosci (2008) 15:757-63  **REPORT** | 76 | WCK | High CPK | Ex 45-55 | Weakness | Negative | 45 |
| Traverso M et al.  Clinical and molecular consequences of exon 78 deletion in DMD gene. J Hum Genet (2018) 63:761-4  **REPORT** | 13 | WCK | High CPK | Ex 78 | Myalgia after exertion, mild hypotrophy of shoulder girdle and pectoral muscles | Negative | n.a. |
| Witting N et al.  Deletion of exon 26 of the dystrophin gene is associated with a mild Becker muscular dystrophy phenotype. Acta Myol (2011) 30:182-4  **REPORT** | 23 | WCK | High CPK | Ex 26 | Muscle pain | Negative | n.a. |
|  | 43 | WCK | Normal | Ex 26 | Muscle pain and calf hypertrophy | Negative | n.a. |
| Schwartz M et al.  Deletion of exon 16 of the dystrophin gene is not associated with disease. Hum Mutat (2007) 28:205  **REPORT** | 60 | A | Normal | Ex 16 | Negative | n.a. | n.a. |
| Zatz M et al. Assessing pathogenicity for novel mutation/sequence variants: the value of healthy older individuals. Neuromolecular Med. (2012) 14(4):281-4.  **PAPER** | 44 | A | Normal | Ex 38-44 | Negative | n.a. | 44 |
|  | 56 | A | Normal | Ex 38-44 | Negative | n.a. | 44 |
