# Supplementary material for: DMD deletions underlining mild dystrophinopathies: literature review highlights phenotype-related mutation clusters and provides insights about genetic mechanisms and prognosis
Source: Front Neurol. 2024 Jan 15;14:1288721. doi: 10.3389/fneur.2023.1288721 (PMC10823016; doi:10.3389/fneur.2023.1288721)
Supplement: Supplementary file 2 [file Table_2.docx]

**Supplementary Table 2.** Distribution of *DMD* deletions in patients with A, CK and WCK phenotype. A = Asymptomatic, CK = Isolated HyperCKemia, WCK = mild weakness with or without high CK, (-): individual/patients from the internal database of the UNIFE Medical Genetics Unit.

[1] Tuffery-Giraud S, Béroud C, Leturcq F, Yaou RB, Hamroun D, Michel-Calemard L et al. Genotype-phenotype analysis in 2,405 patients with a dystrophinopathy using the UMD-DMD database: a model of nationwide knowledgebase. *Hum Mutat.* (2009) 30:934-45. doi: 10.1002/humu.20976

[2] Nakamura A, Fueki N, Shiba N, Motoki H, Miyazaki D, Nishizawa H et al. Deletion of exons 3-9 encompassing a mutational hot spot in the DMD gene presents an asymptomatic phenotype, indicating a target region for multiexon skipping therapy. *J Hum Genet* (2016) 61:663-7. doi: 10.1038/jhg.2016.28

[3] Toksoy G, Durmus H, Aghayev A, Bagirova G, Sevinc Rustemoglu B, Basaran S et al. Mutation spectrum of 260 dystrophinopathy patients from Turkey and important highlights for genetic counseling. *Neuromuscul Disord* (2019) 29:601-613. doi: 10.1016/j.nmd.2019.03.012

[4] Vandenhende MA, Bonnet F, Sailler L, Bouillot S, Morlat P, Beylot J. Dilated cardiomyopathy and lipid-lowering drug muscle toxicity revealing late-onset Becker's disease. *Rev Med Interne* (2005) 26:977-9. French. doi: 10.1016/j.revmed.2005.08.012

[5] Collins AL, Leyland KG, Kennedy CR, Robinson D, Spratt HC. An inherited dystrophin deletion without muscle weakness. *J Med Genet* (1994) 31:505. doi: 10.1136/jmg.31.6.505

[6] Gemelli C, Traverso M, Trevisan L, Fabbri S, Scarsi E, Carlini B et al. An integrated approach to the evaluation of patients with asymptomatic or minimally symptomatic hyperCKemia. *Muscle Nerve.* (2022) 65:96-104. doi: 10.1002/mus.27448

[7] Schwartz M, Dunø M, Palle AL, Krag T, Vissing J. Deletion of exon 16 of the dystrophin gene is not associated with disease. *Hum Mutat* (2007) 28:205. doi: 10.1002/humu.9477

[8] Comi GP, Prelle A, Bresolin N, Moggio M, Bardoni A, Gallanti A et al. Clinical variability in Becker muscular dystrophy. Genetic, biochemical and immunohistochemical correlates. *Brain* (1994) 117 ( Pt 1):1-14. doi: 10.1093/brain/117.1.1-a

[9] England SB, Nicholson LV, Johnson MA, Forrest SM, Love DR, Zubrzycka-Gaarn EE et al. Very mild muscular dystrophy associated with the deletion of 46% of dystrophin. *Nature* (1990) 11;343(6254):180-2. doi: 10.1038/343180a0

[10] Palmucci L, Doriguzzi C, Mongini T, Restagno G, Chiadò-Piat L, Maniscalco M. Unusual expression and very mild course of Xp21 muscular dystrophy (Becker type) in a 60-year-old man with 26 percent deletion of the dystrophin gene. *Neurology* (1994) 44(3 Pt 1):541-3. doi: 10.1212/wnl.44.3_part_1.541

[11] Witting N, Duno M, Vissing J. Deletion of exon 26 of the dystrophin gene is associated with a mild Becker muscular dystrophy phenotype. *Acta Myol* (2011) 30:182-4

[12] Melis MA, Cau M, Muntoni F, Mateddu A, Galanello R, Boccone L et al. Elevation of serum creatine kinase as the only manifestation of an intragenic deletion of the dystrophin gene in three unrelated families. *Eur J Paediatr Neurol* (1998) 2:255-61. doi: 10.1016/s1090-3798(98)80039-1

[13] Beggs AH, Hoffman EP, Snyder JR, Arahata K, Specht L, Shapiro F et al. Exploring the molecular basis for variability among patients with Becker muscular dystrophy: dystrophin gene and protein studies. Am J Hum Genet. 1991 Jul;49:54-67. PMID: 2063877

[14] Zatz M, Pavanello Rde C, Lourenço NC, Cerqueira A, Lazar M, Vainzof M. Assessing pathogenicity for novel mutation/sequence variants: the value of healthy older individuals. *Neuromolecular Med* (2012) 14(4):281-4. doi: 10.1007/s12017-012-8186-x

[15] Saengpattrachai M, Ray PN, Hawkins CE, Berzen A, Banwell BL. Grandpa and I have dystrophinopathy?: approach to asymptomatic hyperCKemia. *Pediatr Neurol* (2006) 35:145-9. doi: 10.1016/j.pediatrneurol.2006.01.004

[16] Helderman-van den Enden AT, Straathof CS, Aartsma-Rus A, den Dunnen JT, Verbist BM, Bakker E et al. Becker muscular dystrophy patients with deletions around exon 51; a promising outlook for exon skipping therapy in Duchenne patients. *Neuromuscul Disord* (2010) 20:251-4. doi: 10.1016/j.nmd.2010.01.013

[17] Anthony K, Cirak S, Torelli S, Tasca G, Feng L, Arechavala-Gomeza V et al. Dystrophin quantification and clinical correlations in Becker muscular dystrophy: implications for clinical trials. *Brain*. (2011) 134:3547-59. doi: 10.1093/brain/awr291

[18] Waldrop MA, Yaou RB, Lucas KK, Martin AS, O'Rourke E; FILNEMUS et al. Clinical Phenotypes of DMD Exon 51 Skip Equivalent Deletions: A Systematic Review. *J Neuromuscul Dis.* (2020) 7:217-229. doi: 10.3233/JND-200483

[19] Bosone I, Bortolotto S, Mongini T, Doriguzzi C, Chiadò-Piat L, Ugo I et al. Late onset and very mild course of Xp21 Becker type muscular dystrophy. *Clin Neuropathol* (2001) 20:196-9. PMID: 11594504

[20] Poyatos-García J, Martí P, Liquori A, Muelas N, Pitarch I, Martinez-Dolz L et al. Dystrophinopathy Phenotypes and Modifying Factors in DMD Exon 45-55 Deletion. *Ann Neurol* (2022) 92:793-806. doi: 10.1002/ana.26461

[21] Ferreiro V, Giliberto F, Muñiz GM, Francipane L, Marzese DM, Mampel A et al. Asymptomatic Becker muscular dystrophy in a family with a multiexon deletion. *Muscle Nerve* (2009) 39:239-43. doi: 10.1002/mus.21193

[22] Taglia A, Petillo R, D'Ambrosio P, Picillo E, Torella A, Orsini C et al. Clinical features of patients with dystrophinopathy sharing the 45-55 exon deletion of DMD gene. *Acta Myol* (2015) 34:9-13. PMID: 26155064

[23] Tselikas L, Rodrigues E, Jammal M, Tiev K, Chayet C, Josselin-Mahr L et al. Late onset Becker muscular dystrophy. A case report and literature review. *Rev Med Interne* (2011) 32:181-6. French. doi: 10.1016/j.revmed.2010.10.353

[24] Nakamura A, Yoshida K, Fukushima K, Ueda H, Urasawa N, Koyama J et al. Follow-up of three patients with a large in-frame deletion of exons 45-55 in the Duchenne muscular dystrophy (DMD) gene. *J Clin Neurosci*. (2008) 15:757-63. doi: 10.1016/j.jocn.2006.12.012

[25] Zimowski JG, Pilch J, Pawelec M, Purzycka JK, Kubalska J, Ziora-Jakutowicz K et al. A rare subclinical or mild type of Becker muscular dystrophy caused by a single exon 48 deletion of the dystrophin gene. *J Appl Genet* (2017) 58:343-347. doi: 10.1007/s13353-017-0391-8

[26] Morrone A, Zammarchi E, Scacheri PC, Donati MA, Hoop RC, Servidei S et al. Asymptomatic dystrophinopathy. *Am J Med Genet* (1997) 69:261-7. doi: 10.1002/(sici)1096-8628(19970331)69:3<261::aid-ajmg9>3.0.co;2-o

[27] Traverso M, Assereto S, Baratto S, Iacomino M, Pedemonte M, Diana MC et al. Clinical and molecular consequences of exon 78 deletion in DMD gene. *J Hum Genet*. (2018) 63:761-764. doi: 10.1038/s10038-018-0439-6
